# Cathepsin B ablation alleviates VSMC phenotypic switching by modulating alternative macrophage polarization through the NLRP3 signaling pathway

**DOI:** 10.3389/fcvm.2026.1820619

**Published:** 2026-05-21

**Authors:** Zushun Gong, Mei Long, Lixin Zhang, Chuantong Wang, Weihua Liu

**Affiliations:** 1 Department of Cardiac Care Unit, ZiBo Central Hospital, Zibo, China; 2 Department of Cardiology, ZiBo Central Hospital, Zibo, China

**Keywords:** Cathepsin B, cGAS-STING, intimal hyperplasia, macrophages, NLRP3 inflammasome, vascular smooth muscle cells

## Abstract

**Introduction:**

The classical activation of pro-inflammatory macrophages contributes to neointimal hyperplasia by driving the excessive accumulation of phenotypically switched vascular smooth muscle cells (VSMCs), a process that underlies occlusive disorders such as atherosclerosis and restenosis. However, the impact of Cathepsin B (CTSB) on the regulation of macrophage polarization remains unclear.

**Methods and Results:**

Analysis of the Gene Expression Omnibus (GEO) database revealed a significant upregulation of CTSB in advanced human atherosclerotic plaques. Furthermore, a time-dependent increase in CTSB expression was observed in carotid arteries following vascular injury. At the cellular level, CTSB expression was markedly elevated in pro-inflammatory M1 macrophages but suppressed in resolving M2 macrophages. A loss-of-function approach, utilizing AdshCTSB-transfected bone marrow-derived macrophages (BMDMs), demonstrated that CTSB knockdown promotes a shift in polarization, repressing M1 markers while inducing those characteristic of the M2 phenotype. This CTSB-mediated polarization switch subsequently attenuated the proliferation and migration of VSMCs while promoting their differentiation. Mechanistically, we identified NLRP3 as a direct target of CTSB. Knockdown of CTSB suppressed the NLRP3 inflammasome, an effect mediated through the cGAS-STING signaling pathway. The functional significance of this pathway was confirmed, as the STING agonist DMXAA abolished the polarizing effects of CTSB silencing. *In vivo*, global CTSB-knockout mice (CTSB-KO) exhibited amelioration of wire injury-induced intimal hyperplasia.

**Discussion:**

In conclusion, our findings suggest that CTSB inhibition represents a promising therapeutic strategy for mitigating intimal hyperplasia. This approach operates by favoring alternative macrophage polarization, which in turn attenuates VSMC phenotypic switching, a process that is partially mediated by the inactivation of the cGAS-STING-NLRP3 axis.

## Introduction

Current primary treatments for stenotic and occlusive atherosclerotic lesions include vascular recanalization techniques such as balloon angioplasty, intravascular stenting, endarterectomy, and bypass graft surgery (1, 2). However, inevitable mechanical injuries to the arteries lead to intimal hyperplasia, which can result in restenosis or re-occlusion. Under physiological conditions, mature vascular smooth muscle cells (VSMCs) exhibit a contractile phenotype and low proliferative activity, stabilizing vascular function. However, following vessel injury, physical and inflammatory stimuli trigger a phenotypic switch in VSMCs, characterized by excessive proliferation, migration, and dedifferentiation. This process drives neointimal formation, narrowing the vascular lumen and promoting restenosis (3). The inflammatory response is a well-established mediator of this pathology, characterized by the recruitment and activation of macrophages. Specifically, classically activated M1 macrophages exacerbate the condition by secreting pro-inflammatory cytokines that accelerate VSMC proliferation and migration. In contrast, alternatively activated M2 macrophages exert anti-inflammatory effects that attenuate the VSMC phenotypic switch and mitigate restenosis (4, 5). Therefore, elucidating the mechanisms governing macrophage polarization is crucial for developing novel therapeutic strategies for intimal hyperplasia.

Cathepsin B (CTSB), a proteolytic enzyme, plays a crucial role in various physiological and pathological processes, such as innate immunity, extracellular matrix remodeling, tumorigenesis, inflammation, and apoptosis (6). Its expression is significantly elevated in macrophages, osteoclasts, and various cancer cells (7). Accumulating evidence has established a link between CTSB and cardiovascular pathology, with its expression observed in conditions such as doxorubicin-induced cardiomyopathy, dilated cardiomyopathy, and human carotid plaques (8–13). These observations suggest that CTSB plays an essential role in cardiovascular diseases. CTSB exacerbates doxorubicin-induced cardiotoxicity by promoting cardiomyocyte apoptosis and oxidative stress through the activation of NF-*κ*B signaling (14). Conversely, CTSB knockout mitigates pressure overload-induced cardiac hypertrophy and remodeling via the TNF-α/ASK1/JNK pathway (9), and its levels in peripheral blood lymphocytes may serve as biomarkers for cardiac hypertrophy (15). Furthermore, elevated CTSB levels are associated with an increased risk of cardiovascular events in patients with stable coronary heart disease and correlate with plaque severity and symptomatic presentation (16). In atherosclerosis, CTSB promotes disease progression by disrupting macrophage iron homeostasis and inducing pyroptosis in VSMCs (17). Despite these established roles in cardiovascular processes, the specific function and underlying mechanism of CTSB in macrophage polarization during intimal hyperplasia remain unknown.

In this study, we demonstrated that CTSB expression was upregulated in advanced atherosclerotic plaques in humans and in a murine model of post-injury carotid arteries. Furthermore, CTSB expression positively correlated with pro-inflammatory M1 macrophage markers and inversely correlated with anti-inflammatory M2 markers. Functional studies revealed that CTSB knockdown in bone marrow-derived macrophages (BMDMs) suppressed the M1 phenotype while promoting the M2 phenotype, as indicated by the expression of the respective marker genes. This shift in macrophage polarization subsequently attenuated the phenotypic switching of VSMCs within a co-culture model. Mechanistically, we identified NLRP3 as a direct target of CTSB and established that CTSB knockdown mediates its effects on macrophage polarization through the inhibition of the cGAS-STING-NLRP3 signaling pathway. Moreover, CTSB ablation attenuated intimal hyperplasia following the wire injury. Collectively, our findings identify CTSB inhibition as a promising therapeutic strategy for arterial restenosis, achieved through the regulation of macrophage polarization and subsequent suppression of VSMC phenotypic switching.

## Materials and methods

### Isolation of macrophages and adenoviral transfection

Mouse peritoneal macrophages (MPMs) were harvested from male mice via peritoneal lavage four days post-intraperitoneal injection of 4% thioglycolate. The collected macrophages were enriched and cultured in RPMI-1640 medium supplemented with 10% fetal bovine serum (FBS) and 1% penicillin–streptomycin. BMDMs were isolated from the femurs and tibias of male mice under sterile conditions. Approximately 5 × 10⁷ nucleated bone marrow cells were cultured in 10 mL of RPMI-1640 medium containing 10% FBS and 50 ng/mL macrophage colony-stimulating factor (M-CSF) for 3 days to induce differentiation. To achieve CTSB knockdown, recombinant adenoviruses expressing CTSB-specific short hairpin RNA (AdshCTSB) were constructed using the GV493 vector, with a non-targeting shRNA adenovirus (AdshNC) serving as the control. BMDMs were infected with the respective adenovirus. Subsequently, for polarization studies, the infected BMDMs were first serum-starved for 24 h and then stimulated with either 100 ng/mL lipopolysaccharide (LPS) to induce an M1 phenotype or 10 ng/mL interleukin-4 (IL-4) to induce an M2 phenotype.

### Analysis of VSMC proliferation and migration

The conditioned medium was collected from BMDMs (1 × 10^6^ cells/well in a six-well plate) that were transfected with either AdshRNA or AdshCTSB. This was performed following stimulation for 24 h with LPS or IL-4, and the cells were cultured in Dulbecco's Modified Eagle’s Medium (DMEM) supplemented with 10% FBS and antibiotics. The conditioned medium was filtered through a 0.22 µm filter and diluted 1:1 with fresh medium. Primary VSMCs were isolated from the thoracic aorta of male mice through enzymatic digestion. After a 24-h serum starvation period, VSMCs were seeded at a density of 1 × 10^4^ cells/well into a 96-well plate and co-cultured with the conditioned medium. The ratio of VSMCs to macrophages was approximately 1:2. VSMC proliferation was assessed using a BrdU incorporation assay, with BrdU introduced during the final 2 h of the co-culture period. The level of incorporated BrdU was quantified using a commercial cell proliferation BrdU kit, according to the manufacturer's protocol. VSMC migration was evaluated using a transwell chamber assay, wherein serum-starved VSMCs in 200 µL of serum-free medium were placed in the upper chamber, and the corresponding conditioned medium from BMDMs was added to the lower chamber. After 24 h of incubation, cells that migrated to the underside of the filter were fixed with 4% paraformaldehyde, stained with 0.1% crystal violet, and then counted.

### Quantitative real-time PCR and western blot analysis

Total RNA was extracted from the carotid artery tissues or cultured cells using TRIzol reagent. Subsequently, 2 µg of total RNA was reverse-transcribed into complementary DNA (cDNA). Quantitative real-time PCR (qRT-PCR) was conducted using a LightCycler 480 II system with specific primers targeting the genes of interest. For western blot analysis, total protein was extracted from cells using RIPA lysis buffer, and its concentration was quantified using a bicinchoninic acid assay kit. Equal amounts of protein were separated by sodium dodecyl sulfate - polyacrylamide gel electrophoresis and then transferred onto a polyvinylidene fluoride membrane. The membrane was blocked with 5% non-fat milk and subsequently incubated with specific primary antibodies overnight at 4 °C. Following incubation with the appropriate horseradish peroxidase-conjugated secondary antibodies for 1 h at room temperature, the protein bands were visualized using a Fluor Chem E imager. The protein expression levels were normalized to glyceraldehyde-3-phosphate dehydrogenase.

### Animals and treatment

All animal study protocols were approved by the Institutional Animal Care and Use Committee of the ZiBo Central Hospital. CTSB^−/+^ heterozygous mice were purchased from Jackson Laboratory. CTSB knockout (CTSB-KO) mice and their wild-type (WT) littermates were anesthetized with sodium pentobarbital administered intraperitoneally, after which the left carotid artery was carefully dissected. The segment proximal to the bifurcation point of the external carotid artery was ligated with sutures, and vascular clamps were used to interrupt blood flow in the internal and common carotid arteries. A guidewire was introduced into the arterial lumen and withdrawn five times in a rotating motion following a transverse incision. The mice were sacrificed at 7 and 14 days post-injury. Consecutive 3-μm sections of the entire region were obtained from the bifurcation site of the left carotid artery, and hematoxylin and eosin (HE) staining was utilized for morphometric analysis.

### Statistical analysis

All data are presented as mean ± standard deviation (SD). Statistical analyses were performed using the SPSS software (version 19.0). For parametric statistical analysis between the two groups, a two-tailed Student's t-test was employed. A one-way ANOVA was conducted for comparisons among more than two groups, followed by either a Bonferroni *post-hoc test* or Tamhane's T2 *post-hoc test*. A *P*-value of less than 0.05 was considered statistically significant.

## Results

### CTSB expression is upregulated in atherosclerotic plaques, injured arteries, and pro-inflammatory macrophages

**Figure 1:**
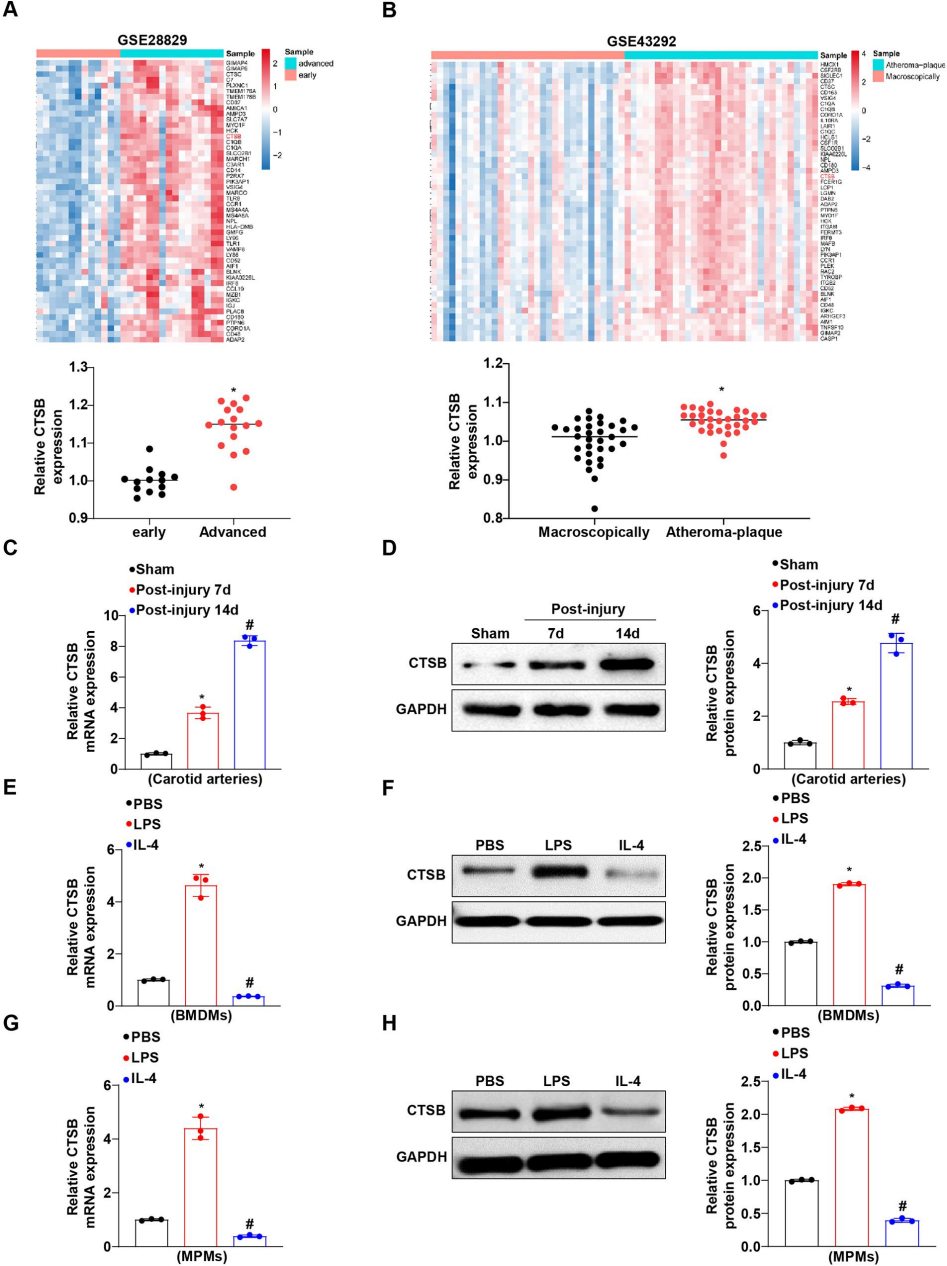
CTSB expression is induced in the neointima and M1 macrophages. **(A,B)** Volcano plots of publicly available data from the GEO databases of GSE28829 and GSE43292. The expression of CTSB in advanced atherosclerotic plaques compared to early-stage plaques (GSE28829), and atheromatous plaques versus macroscopic tissue (GSE43292). *P < 0.05 versus the control group. **(C,D)** Western blot analysis of CTSB protein levels in carotid arteries followed the injury time at 7 and 14 days. *P < 0.05 versus the sham group. #*P* < 0.05 versus the post-injury at 7-day group. **(E,F)** Relative mRNA and protein levels of CTSB in BMDMs upon LPS or IL-4 stimulation. *P < 0.05 versus the PBS group. #*P* < 0.05 versus the LPS group. **(G,H)** Relative mRNA and protein levels of CTSB in MPMs upon LPS or IL-4 stimulation. *P < 0.05 versus the PBS group. #*P* < 0.05 versus the LPS group.

To investigate the role of CTSB in intimal hyperplasia and atherosclerosis, we first analyzed its expression in both human diseases and experimental models. An examination of publicly available Gene Expression Omnibus (GEO) datasets (GSE28829 and GSE43292) revealed that CTSB expression was significantly elevated in advanced human atherosclerotic plaques compared to early lesions (Figure 1A,B). To model this process, we induced neointimal hyperplasia in mice by wire-mediated carotid artery injury. Consistent with the human data, both RT-PCR and western blot analyses demonstrated a time-dependent upregulation of CTSB expression in the injured carotid arteries at days 7 and 14 post-injury (Figure 1C,D). Given the central role of macrophage polarization in driving intimal hyperplasia, we assessed CTSB expression in polarized macrophages. In both BMDMs and MPMs, stimulation with LPS (an M1 inducer) significantly upregulated CTSB expression, while IL-4 (an M2 inducer) suppressed its expression (Figures 1E–H). Collectively, these findings establish a close association between CTSB upregulation and pro-inflammatory macrophage activation in vascular pathology.

### CTSB knockdown attenuates M1 and promotes M2 macrophage polarization

**Figure 2:**
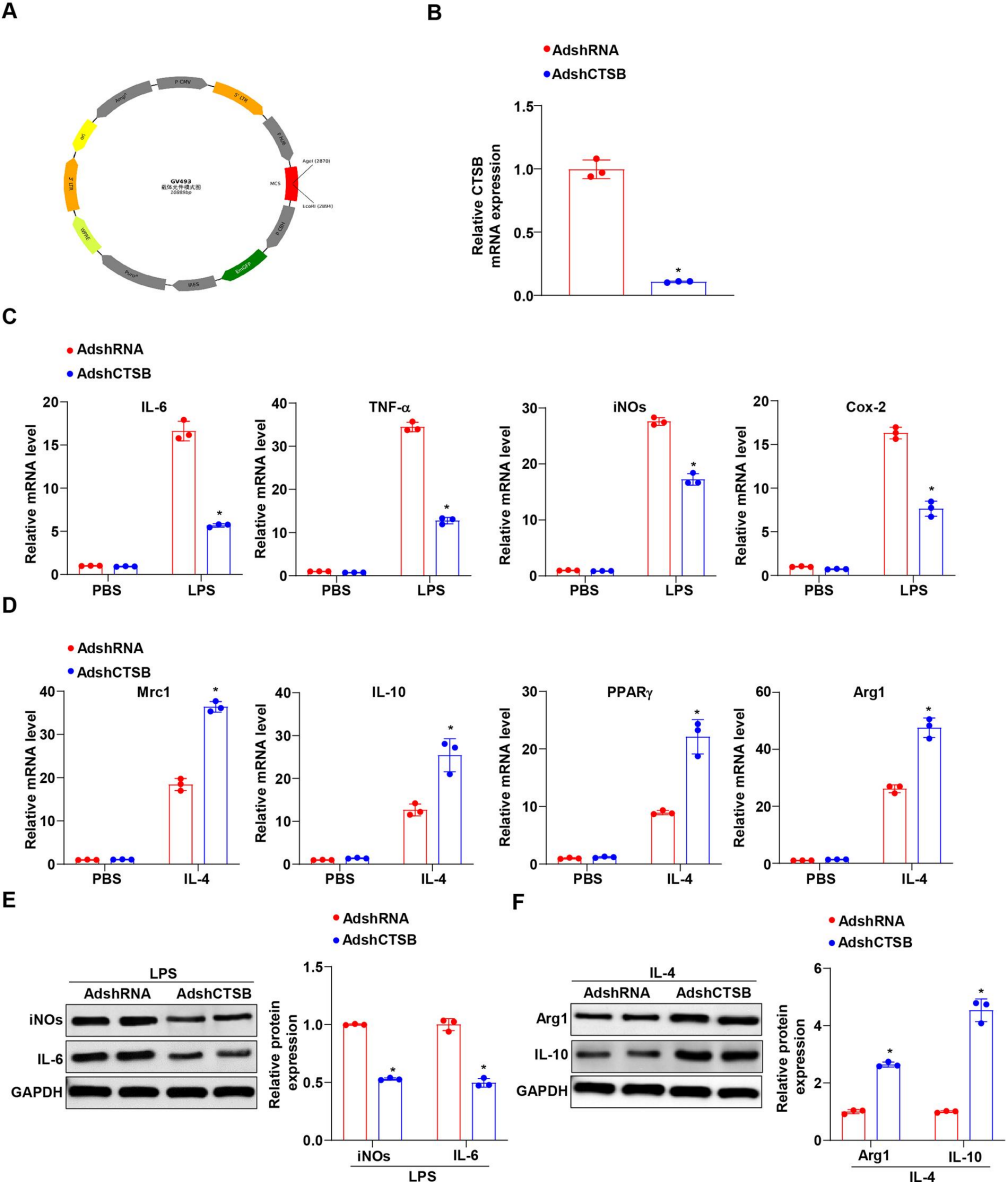
CTSB knockdown promotes M2 polarization of macrophages *in vitro*. **(A)** Generation of AdshCTSB adenoviruses using the GV493 vector. **(B)** mRNA expression of CTSB in BMDMs transfected with AdshCTSB and AdshRNA. **(C)** RT-PCR of markers related to pro-inflammatory M1 macrophages, including IL-6, TNF-α, iNOs, and Cox-2 in BMDMs transfected with AdshCTSB and AdshRNA and treated with PBS or LPS. **(D)** RT-PCR of markers related to anti-inflammatory M2 macrophages, including Mrc1, IL-10, PPAR*γ*, and Arg1 in BMDMs transfected with AdshCTSB and AdshRNA and treated with PBS or IL-4. **(E)** Western blot analysis of iNOs and IL-6 protein levels in BMDMs transfected with AdshCTSB and AdshRNA and treated with PBS or LPS. **(F)** Western blot analysis of Arg1 and IL-10 protein levels in BMDMs transfected with AdshCTSB and AdshRNA and treated with PBS or IL-4. *P < 0.05 versus the AdshRNA group.

To evaluate the role of CTSB in macrophage polarization, we transfected BMDMs with an AdshCTSB and subsequently stimulated them with LPS or IL-4 (Figure 2A). The efficiency of CTSB knockdown in BMDMs was confirmed by RT-PCR analysis (Figure 2B). We observed that LPS stimulation led to a reduction in the mRNA expression of prototypical M1 macrophage-associated genes, including IL-6, TNF-α, iNOS, and COX-2, in BMDMs with AdshCTSB compared to the AdshRNA group, which exhibited no significant differences when treated with PBS (Figure 2C). Conversely, the results indicated that IL-4-induced markers associated with M2 polarization, including Mrc1, IL-10, PPAR*γ*, and Arg1, were significantly upregulated in BMDMs treated with AdshCTSB compared to the controls (Figure 2D). Consistent with the mRNA results, a similar effect of AdshCTSB on the protein levels of M1 (iNOS and IL-6) and M2 (Arg1 and IL-10) marker genes was observed (Figures 2E,F). These data suggest that CTSB knockdown promotes the alternative activation of macrophages.

### CTSB overexpression enhances M1 and inhibits M2 macrophage polarization

**Figure 3:**
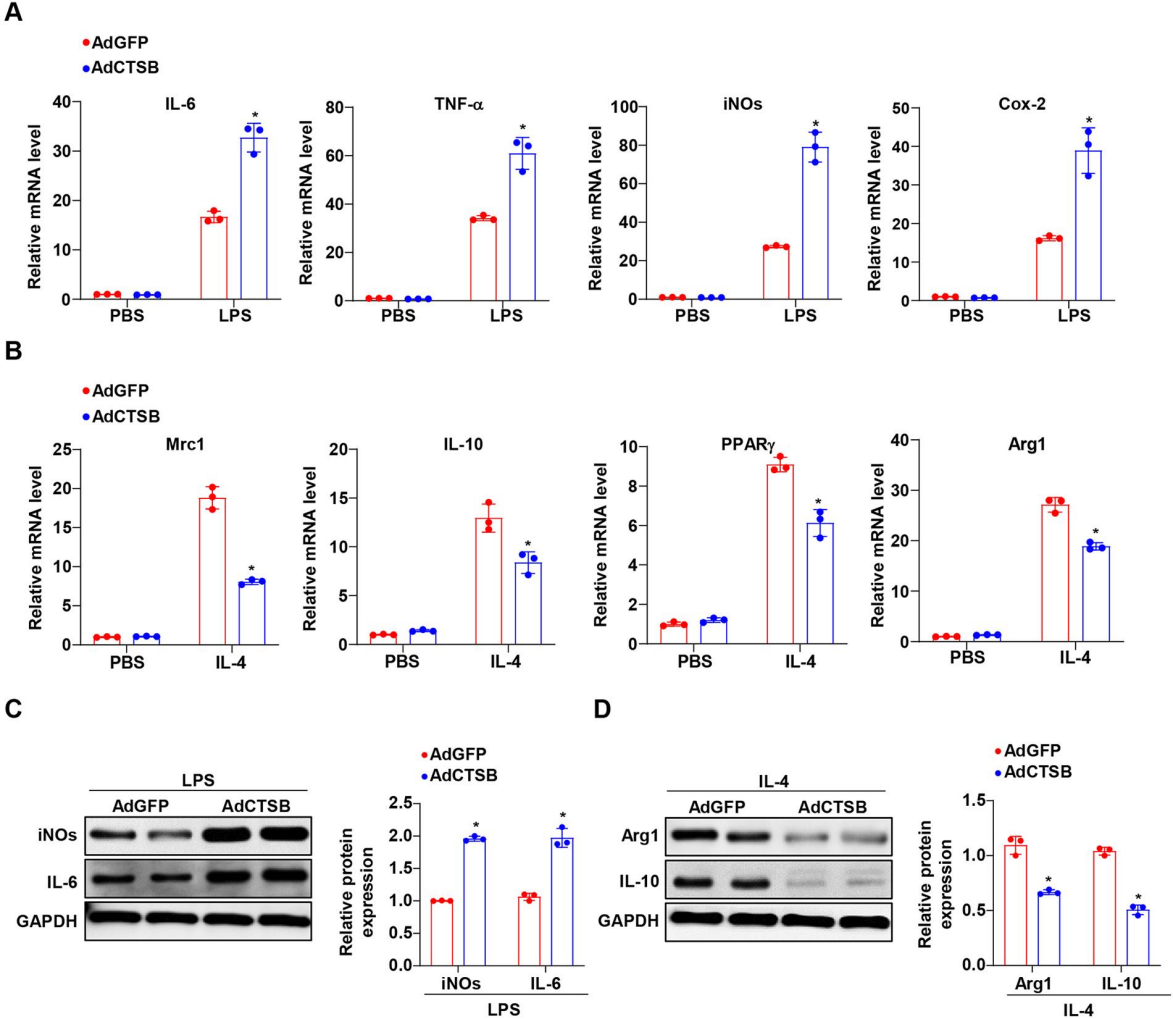
CTSB overexpression promotes M1 polarization of macrophages *in vitro*. **(A)** RT-PCR of markers related to pro-inflammatory M1 macrophages, including IL-6, TNF-α, iNOs, and Cox-2 in BMDMs transfected with AdCTSB and AdGFP and treated with PBS or LPS. **(B)** RT-PCR of markers related to anti-inflammatory M2 macrophages, including Mrc1, IL-10, PPAR*γ*, and Arg1 in BMDMs transfected with AdCTSB and AdGFP treated with PBS or IL-4. **(C)** Western blot analysis of iNOs and IL-6 protein levels in BMDMs transfected with AdCTSB and AdGFP and treated with PBS or LPS. **(D)** Western blot analysis of Arg1 and IL-10 protein levels in BMDMs transfected with AdCTSB and AdGFP and treated with PBS or IL-4. *P < 0.05 versus the AdGFP group.

Next, we examined the role of CTSB overexpression by transfecting BMDMs with an adenovirus harboring Cathepsin B RNA (AdCTSB) and subsequently administering LPS or IL-4 to assess macrophage polarization. We observed that the mRNA expression of prototypical M1 macrophage-associated genes, including IL-6, TNF-α, iNOS, and COX-2, was significantly increased in BMDMs transfected with AdCTSB compared to that in the AdGFP group subjected to LPS stimulation. Notably, no significant differences were observed when treated with PBS (Figure 3A). Conversely, markers associated with M2 polarization, including Mrc1, IL-10, PPAR*γ*, and Arg1, were significantly downregulated in BMDMs transfected with AdCTSB compared to those induced by IL-4 treatment (Figure 3B). Consistent with the mRNA results, similar trends were observed in the protein levels of M1 (iNOS and IL-6) and M2 (Arg1 and IL-10) marker genes following AdCTSB treatment (Figures 3C,D). These data suggest that CTSB overexpression promotes the classical activation of macrophages.

### The crosstalk between macrophage polarization mediated by CTSB knockdown and VSMCs

**Figure 4:**
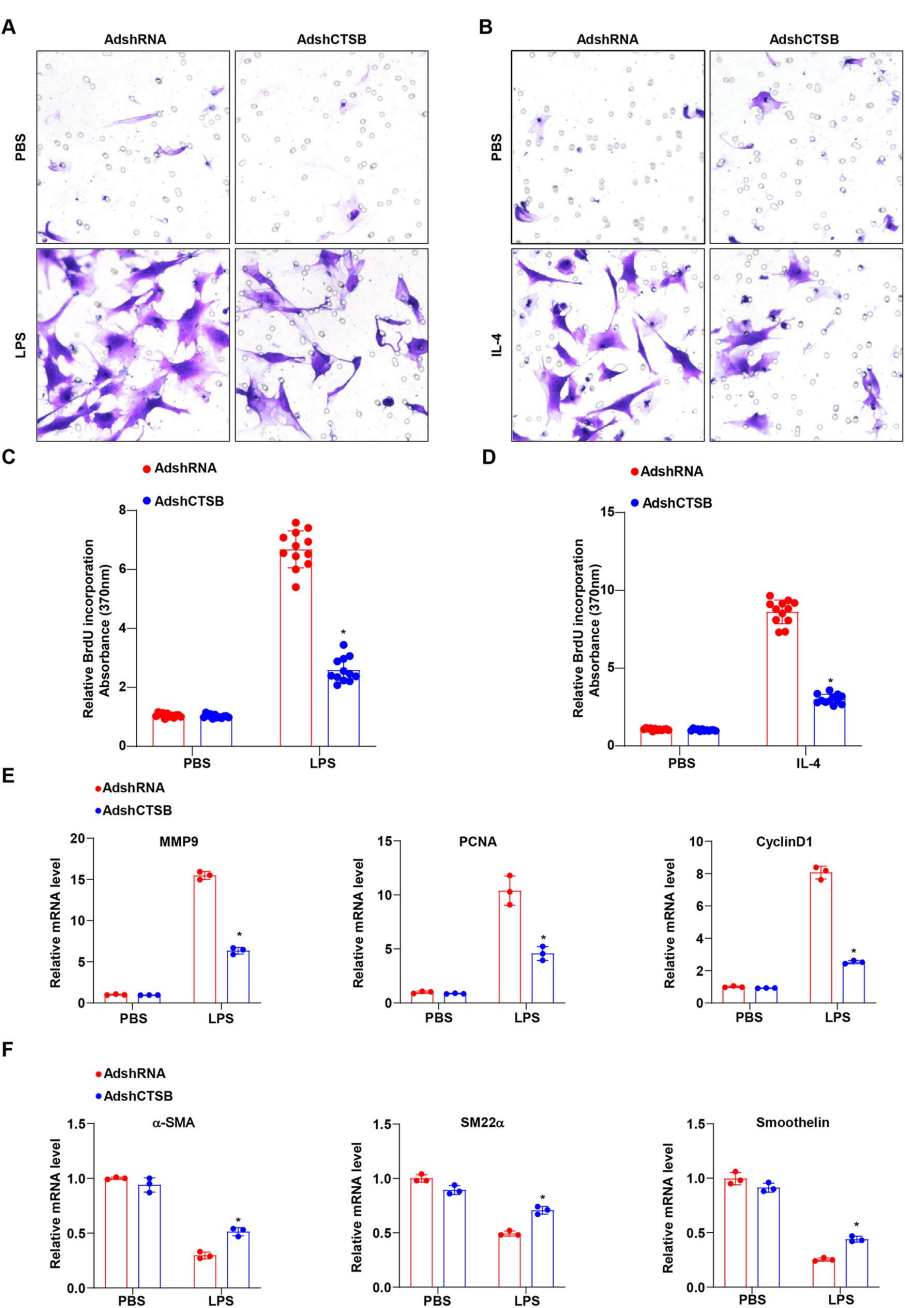
Effect of macrophage polarization mediated by AdshCTSB on VSMCs phenotypic switching. **(A,B)** The number of migrated VSMCs detected using the transwell migration assay induced by co-culturing with BMDMs transfected with AdshCTSB and AdshRNA and treated with LPS **(A)** or IL-4 **(B)**. **(C,D)** BrdU incorporation was determined by measuring the absorption at 370 nm to assess VSMCs proliferation in conditional medium isolated from cultured BMDMs transfected with AdshCTSB and AdshRNA upon LPS treatment **(C)** or IL-4 treatment **(D)**. **(E,F)** RT-PCR analysis of the representative mRNA expression of migration (MMP9), proliferation (PCNA and CyclinD1), but increased the expression of differentiation markers of VSMCs (*α*-SMA, SM22*α,* and Smoothelin) after co-culturing with conditional medium isolated from cultured BMDMs transfected with AdshCTSB and AdshRNA before or after LPS treatment. *P < 0.05 versus the AdshRNA group with LPS or IL-4 treatment.

Next, we aimed to further investigate the effect of CTSB knockdown on the phenotypic switching of VSMCs, which is characterized by increased proliferation, migration, and dedifferentiation. We collected conditioned medium from BMDMs transfected with AdshCTSB or AdshRNA, following stimulation with LPS or IL-4. The supernatants were then used for direct co-culture with VSMCs, or VSMCs were placed in the upper chamber of a Transwell assay. We utilized the Transwell chamber and BrdU assays to measure the migration and proliferation abilities of VSMCs influenced by CTSB knockdown. The results demonstrate a moderate decrease in cell invasion and proliferative capacity when exposed to LPS-conditioned media from BMDMs transfected with AdshCTSB (Figures 4A,C), while more pronounced effects were observed with IL-4 stimulation (Figures 4B,D). Furthermore, the results indicate that CTSB silencing significantly reduced the mRNA expression levels of migration markers (MMP9) and proliferation markers (PCNA and Cyclin D1) (Figure 4E), while increasing the expression of differentiation markers in VSMCs (*α*-SMA, SM22*α*, and Smoothelin) when co-cultured with the medium collected from M1 polarized macrophages (Figure 4F). Collectively, these findings suggest that CTSB plays a significant role in mediating the crosstalk between macrophage polarization and phenotypic switching of VSMCs.

### CTSB knockdown inhibits the cGAS-STING-NLRP3 signaling pathway

**Figure 5:**
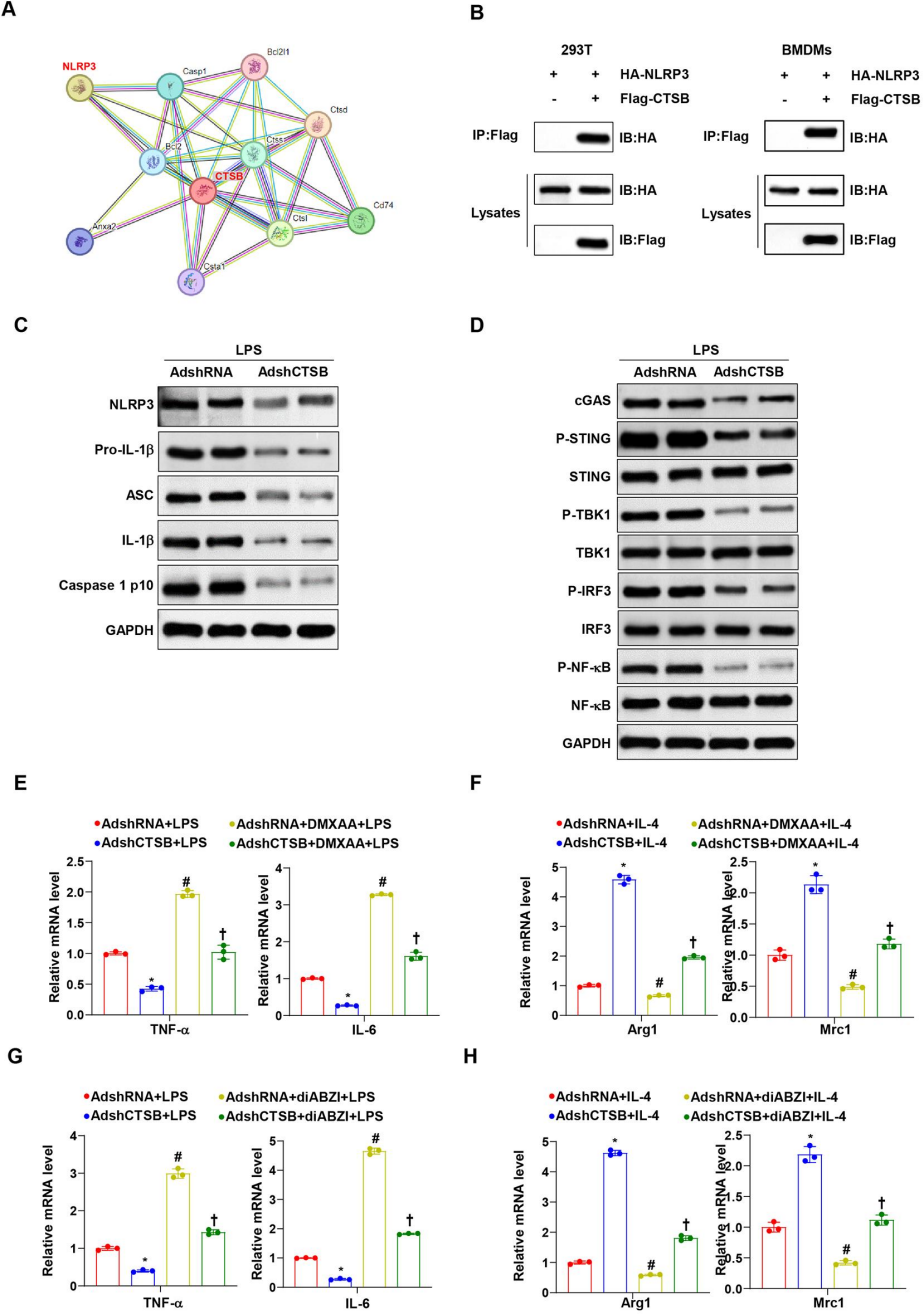
CTSB knockdown inhibits the cGAS-STING-NLRP3 signaling pathway. **(A)** Potential targets for CTSB analysis from the string network. **(B)** Representative western blots of Co-IP assays in 293 T cells transfected with Flag-tagged CTSB and HA-tagged NLRP3. Flag and HA antibodies were used as western blotting probes. **(C)** Western blot analysis of the expression of NLRP3 inﬂammasome-associated proteins in BMDMs transfected with AdshCTSB and AdshRNA with LPS stimulation. **(D)** Western blot analysis of the expression of cGAS, p-STING/STING, P-TBK1/TBK1, and P-IRF3/IRF3 in BMDMs transfected with AdshCTSB and AdshRNA with LPS stimulation. **(E,F)** RT-PCR analysis of TNF-α and IL-6 for pro-inflammatory macrophages, Arg1, and Mrc1 for anti-inflammatory macrophages in BMDMs transfected with AdshCTSB and AdshRNA stimulated with or without DMXAA and subjected to LPS administration. **(G,H)** RT-PCR analysis of TNF-α and IL-6 for pro-inflammatory macrophages, Arg1, and Mrc1 for anti-inflammatory macrophages in BMDMs transfected with AdshCTSB and AdshRNA stimulated with or without diABZI and subjected to LPS administration. *P < 0.05 versus the control group upon LPS stimulation. #*P* < 0.05 versus AdshCTSB group upon LPS stimulation. †*P* < 0.05 versus the control group stimulated with DMXAA and LPS stimulation.

To investigate the mechanisms underlying the effects of CTSB knockdown on anti-inflammatory macrophage regulation, we utilized a string network to identify potential targets. We observed that CTSB interacts with NLRP3 in 293 T cells and BMDMs, a finding further validated by immunoprecipitation (IP) of CTSB and NLRP3 (Figures 5A,B). Given the established role of the NLRP3 inflammasome in inflammatory diseases, we examined the protein expression of NLRP3 inflammasome components mediated by CTSB. The results indicate reduced levels of NLRP3-associated proteins (NLRP3, pro-IL-1β, ASC, IL-1β, and caspase-1 p10) in LPS-treated BMDMs transfected with AdshCTSB (Figure 5C). Accumulating evidence implicates the cGAS-STING pathway in the triggering and activation of the NLRP3 inflammasome, a connection that is particularly relevant in the pathogenesis of cardiovascular diseases. Furthermore, we detected impaired activation of the cGAS-STING-TBK1-IRF3/NF-*κ*B pathway in BMDMs transfected with AdshCTSB upon LPS stimulation (Figure 5D). Functionally, the STING agonist DMXAA mitigated the decreased expression of M1 (TNF-α and IL-6) and enhanced the expression of M2 (Arg-1 and Mrc1) polarized macrophage markers due to CTSB knockdown (Figures 5E,F). Similar results were also obtained with another STING agonist, diABZI (Figures 5G,H). Moreover, we observed that the increased M1 and decreased M2 relative marker expression was abolished by using the pharmacological NLRP3 inhibitor MCC950 (Supplementary Materials). Thus, these findings suggest that the effects of CTSB knockdown on macrophage polarization are partially mediated by inactivation of the cGAS-STING-NLRP3 signaling pathway.

### CTSB ablation attenuates arterial intimal hyperplasia

**Figure 6:**
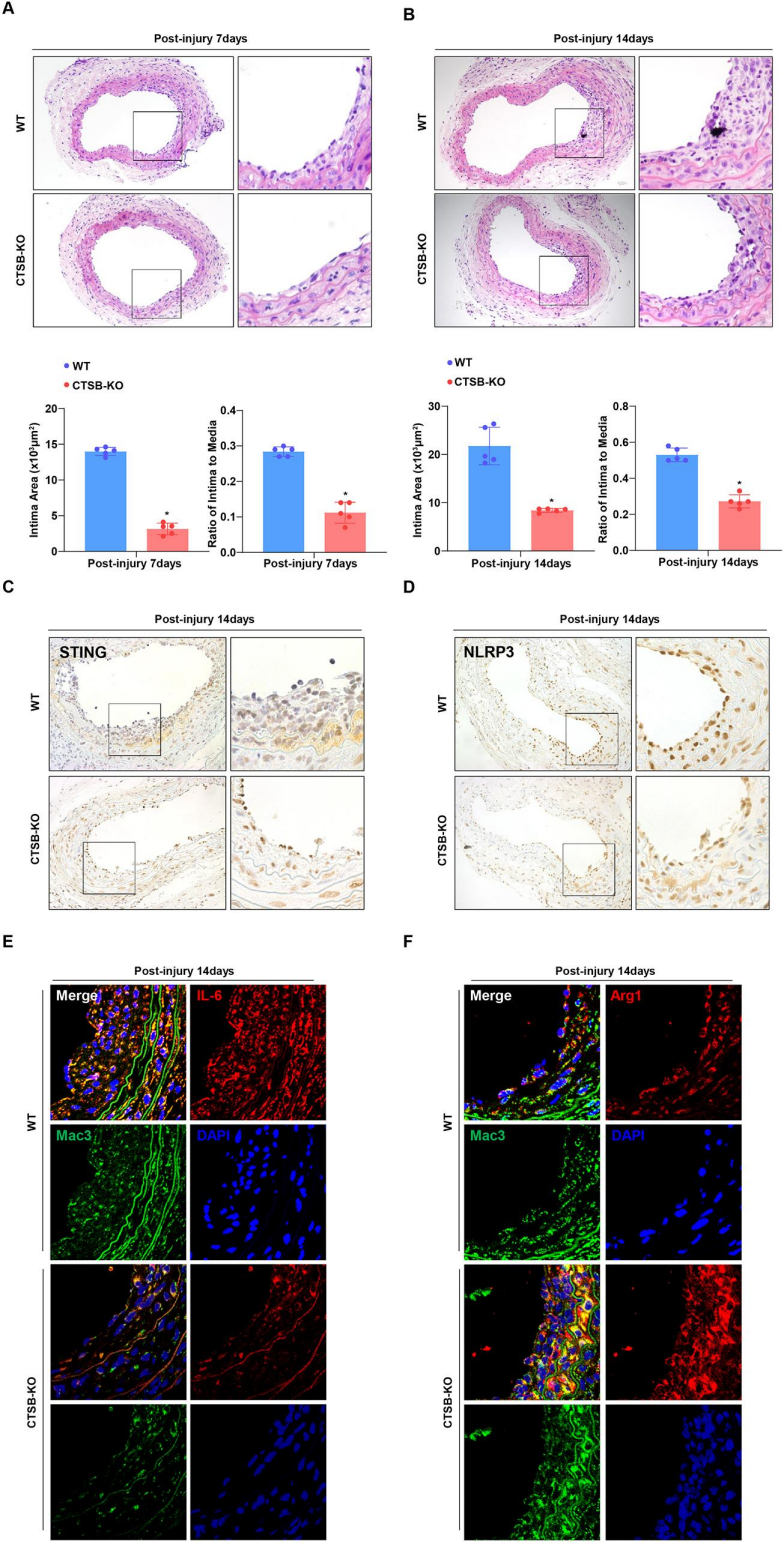
CTSB deficiency modulated intimal hyperplasia. **(A,B)** HE-stained sections demonstrating the intimal areas of WT and CTSB-KO mice at the indicated times. Intimal areas and intima/media ratios were quantiﬁed in the bottom panel. **(C,D)** Representative immunohistochemical images of STING and NLRP3 in neointimal sections of CTSB ablation mice compared with WT mice. *P < 0.05 versus the WT group. **(E,F)** Double immunofluorescence staining of IL-6 or Arg-1 with Mac3 in the sections of the neointima 14 days after neointima formation in WT and CTSB-KO mice. *P < 0.05 versus the WT group.

Given the predominant expression of CTSB in neointimal macrophages and its role in macrophage polarization *in vitro*, we investigated the impact of CTSB deficiency on intimal hyperplasia. To test this hypothesis, we performed carotid artery wire injury surgery on CTSB-KO mice and WT controls. The neointima in CTSB-KO mice was significantly thinner than that in WT mice. In addition, the intima-to-media (I/M) ratio in the arteries of CTSB-KO mice was significantly lower than that of WT controls at both 7 and 14 days post-injury (Figures 6A,B). Furthermore, reduced expression of STING and NLRP3 was observed in the neointima of CTSB-KO mice compared to that in WT controls at 14 days post-injury (Figures 6C,D). Moreover, immunofluorescence staining of the carotid artery neointima tissue sections indicated that IL-6 was less abundant in the neointimal macrophages of CTSB-KO mice than in those of WT mice at 14 days after injury (Figure 6E). In contrast, Arg-1 expression in the macrophages of the CTSB-KO carotid artery neointima was significantly higher than that in the controls (Figure 6F).

## Discussion

Despite significant advancements in vascular revascularization techniques, the treatment of arterial restenosis remains constrained by intimal hyperplasia, a process predominantly driven by macrophage accumulation and the subsequent phenotypic switching of VSMCs. In this study, we identified a novel role for CTSB in this pathophysiology. We demonstrated that the knockdown of CTSB shifted the macrophage equilibrium toward a restorative M2 phenotype, which in turn attenuated the pathological proliferation, migration, and dedifferentiation of VSMCs. Mechanistically, we established that CTSB directly targets NLRP3 and that its silencing suppresses the cGAS-STING signaling pathway, thereby mediating its regulatory effects on macrophage polarization. These findings suggest that CTSB inhibition is a promising therapeutic strategy for combating intimal hyperplasia.

CTSB is a proteolytic enzyme implicated in various pathological processes, including innate immunity, extracellular matrix remodeling, inflammation, apoptosis, oxidative stress, and oncogenesis (7, 18). Its significant role in cardiovascular diseases is highlighted by its upregulated expression in pathological cardiomyocytes, human carotid plaques, and stromal tissues (10, 12–14, 16). Functionally, CTSB exacerbates doxorubicin-induced cardiotoxicity by promoting cardiomyocyte apoptosis and oxidative stress through the activation of NF-*κ*B signaling (14). Conversely, genetic knockout of CTSB mitigates pressure overload-induced cardiac hypertrophy and remodeling via the TNF-α/ASK1/JNK pathway (9). Furthermore, elevated CTSB levels are associated with an increased risk of cardiovascular events in patients with stable coronary heart disease and correlate with plaque severity and symptomatology (12, 16, 19). In the vasculature, CTSB promotes apoptosis in endothelial and VSMCs, and foam cell formation, thus accelerating atherogenesis (15, 17). A critical step in this process is endothelial dysfunction, which drives the secretion of chemokines and adhesion molecules that facilitate monocyte infiltration and their subsequent differentiation into macrophages (19). These macrophages then secrete a milieu of cytokines and growth factors that promote VSMC phenotypic switching, a key event in neointima formation and atherosclerosis (20). Building on the established role of CTSB in vascular inflammation, the present study provides novel mechanistic insights. We found that CTSB expression was significantly upregulated in macrophages upon LPS stimulation (M1 polarization) but was downregulated following IL-4 treatment (M2 polarization). Macrophages produce paracrine signals via multiple lipid metabolic pathways, including *de novo* fatty acid synthesis, fatty acid oxidation, and cholesterol metabolism. These signals, in the form of lipid mediators or cytokines, regulate the proliferation, activation, and phenotypic switching of fibroblasts and VSMCs, thereby participating in the pathological processes of cardiovascular diseases, such as myocardial infarction and atherosclerosis. Functionally, CTSB knockdown shifted macrophage equilibrium toward the M2 phenotype, as evidenced by altered marker gene expression. This shift in polarization, in turn, dramatically attenuates the pathological proliferation, migration, and dedifferentiation of VSMCs. The potential effect of CTSB on macrophage metabolic states may also serve as a mechanistic link underlying the changes in VSMCs' behavior mediated by CTSB.

Aberrant activation of the NLRP3 inflammasome is a well-documented driver of numerous inflammatory diseases and is recognized as a critical mediator of conditions such as cryopyrin-associated periodic syndromes, type 2 diabetes, obesity, and atherosclerosis. Its activation, triggered by diverse pathogen-, environmental-, and host-derived factors, initiates the assembly of a multimolecular complex: NLRP3 binds to the adaptor protein ASC (apoptosis-associated speck-like protein containing a CARD), which recruits and activates the cysteine protease caspase-1. Active caspase-1 subsequently cleaves pro-IL-1β and pro-IL-18 into mature, bioactive forms, amplifying the inflammatory cascade. The NLRP3 inflammasome plays a particularly significant role in the pathophysiology of the cardiovascular system. Its core components-NLRP3, ASC, caspase-1, IL-1β, and IL-18-are highly expressed in human carotid atherosclerotic plaques, and their levels correlate with disease severity. Beyond its role in innate immunity, the NLRP3 inflammasome is a key regulator of VSMC behavior, influencing migration, proliferation, and phenotypic transformation (21). Accumulating evidence identifies the cGAS-STING pathway as a crucial upstream regulator of NLRP3 inflammasome activation, a link of significant relevance to cardiovascular pathogenesis (22). The primary mechanism by which cGAS-STING exerts its inflammatory effects is by directing macrophage polarization (23). Macrophage activation exhibits remarkable plasticity in regulating inflammation, as differentiated macrophages can switch between the classical (M1) phenotype, characterized by the production of cytokines such as TNF-α, IL-1β, and IL-12, while simultaneously suppressing the alternative M2 anti-inflammatory phenotype via the inhibition of STAT6 signaling. Notably, anti-inflammatory M2 macrophages possess an enhanced efferocytic capacity, which plays a critical role in various physiological and pathological processes, including tissue repair, inflammation resolution, and regeneration. Previous studies have demonstrated that CTSB promotes pyroptosis in human umbilical vein endothelial cells by activating the NLRP3 inflammasome (24). In addition, it leads to caspase-1 activation and pyroptosis in BMDMs through its interaction with NLRP3 at the endoplasmic reticulum level (25, 26). Furthermore, CTSB promotes VSMC pyroptosis in atherosclerotic plaques, exacerbating plaque progression by increasing the expression of NLRP3 (17). Building on this foundation, our study provides a unifying mechanistic insight: we demonstrate that CTSB directly interacts with NLRP3 and that its regulation of macrophage polarization is mediated through the cGAS-STING-NLRP3 signaling axis. A key feature of mitochondrial injury is the release of mitochondrial DNA (mtDNA) into the cytosol. As a DNA sensor, cGAS detects both self-DNA and non-self-DNA, thereby initiating cellular injury responses. Upon binding to cytosolic double-stranded DNA (dsDNA), cGAS catalyzes the synthesis of cyclic GMP–AMP (cGAMP) from adenosine triphosphate and guanosine triphosphate. The second messenger cGAMP subsequently binds to STING, which serves as a critical activator of pro-inflammatory transcriptional programs. In addition to the cGAS-STING axis, mitochondrial respiration also regulates inflammatory cytokine production and immune cell function. Recent studies have demonstrated that impairing mitochondrial respiration by genetically depleting Tfam, a gene essential for mitochondrial DNA stability, suppresses inflammation in macrophages (27). Furthermore, SerpinB2 enhances the survival of adipose tissue-resident macrophages by regulating mitochondrial oxidative phosphorylation and preventing the release of pro-apoptotic cytochrome c from the mitochondria into the cytoplasm through antioxidant glutathione production (28). In addition, GSDMD contributes to mitochondrial perforation and mtDNA leakage, acting upstream of the STING-IRF3/NF-*κ*B axis to inhibit cytokine secretion and the development of atherosclerosis (29). Given the established connections between mitochondrial integrity, the cGAS-STING pathway, and macrophage function, we considered the potential role of CTSB, a lysosomal cysteine protease, in regulating the cGAS-STING pathway during macrophage polarization.

In conclusion, our findings demonstrate that CTSB is upregulated in human atherosclerotic plaques and experimental neointimal hyperplasia, and its expression is positively correlated with pro-inflammatory M1 macrophage polarization. Through a loss-of-function approach, we clarified that the knockdown of CTSB shifted the macrophage equilibrium toward an anti-inflammatory M2 phenotype, an effect that subsequently attenuated the pathological phenotypic switching of VSMCs *in vitro*. Mechanistically, we established that CTSB directly targets NLRP3, and its silencing suppresses the NLRP3 inflammasome, with the regulation of macrophage polarization mediated through the cGAS-STING signaling pathway. Collectively, this study identifies CTSB as a novel and promising therapeutic target for treating arterial restenosis by modulating macrophage polarization via the cGAS-STING-NLRP3 axis.

## Data Availability

The datasets presented in this study can be found in online repositories. The names of the repository/repositories and accession number(s) can be found in the article/Supplementary Material.
