# Supplementary figures and images for: Cathepsin B ablation alleviates VSMC phenotypic switching by modulating alternative macrophage polarization through the NLRP3 signaling pathway
Source: Front Cardiovasc Med. 2026 May 21;13:1820619. doi: 10.3389/fcvm.2026.1820619 (PMC13233365; doi:10.3389/fcvm.2026.1820619)

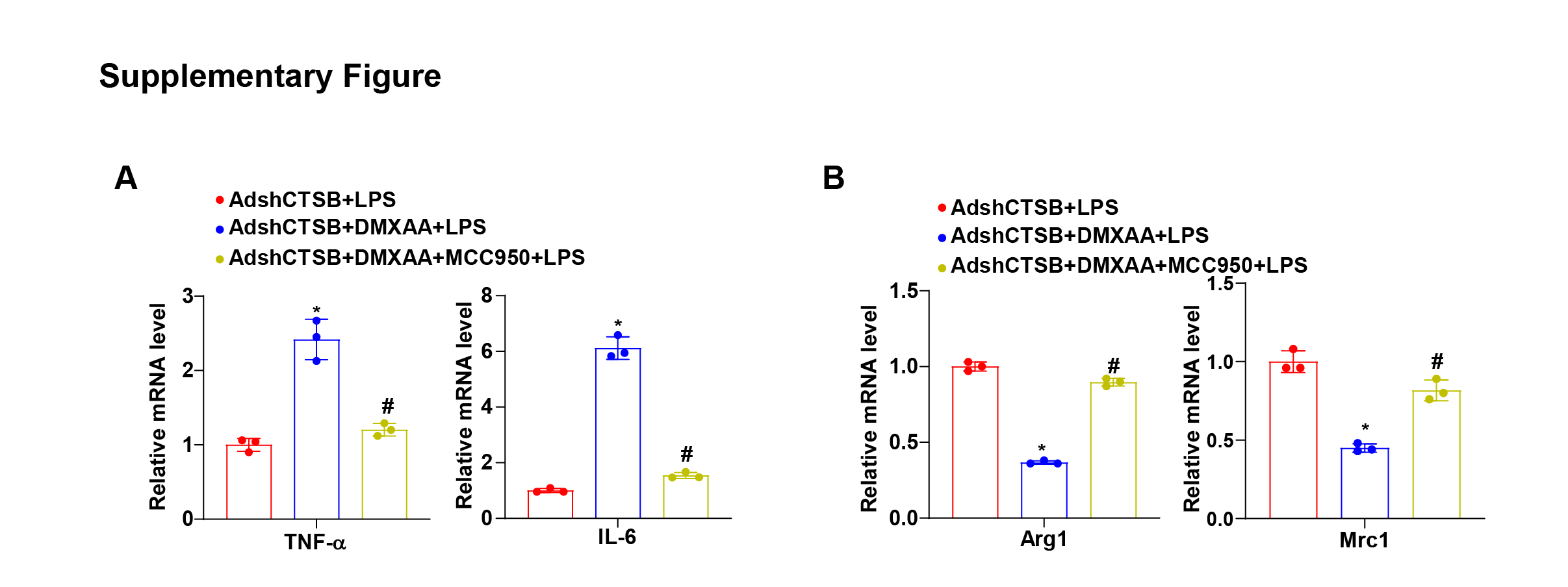

Supplement: Supplementary file 1 [file Image1.jpeg]
